# Endocrine Disruption: Structural Interactions of Androgen Receptor against Di(2-ethylhexyl) Phthalate and Its Metabolites

**DOI:** 10.3390/toxics8040115

**Published:** 2020-12-08

**Authors:** Mohd Amin Beg, Ishfaq Ahmad Sheikh

**Affiliations:** 1 King Fahd Medical Research Center, King Abdulaziz University, Jeddah 21589, Saudi Arabia; iasheikh@kau.edu.sa; 2 Department of Medical Laboratory Technology, Faculty of Applied Medical Sciences, King Abdulaziz University, Jeddah 21589, Saudi Arabia

**Keywords:** androgen receptor, DEHP, MEHP, 5-OH-MEHP, 5-oxo-MEHP, 5-cx-MEPP, 2-cx-MMHP, docking

## Abstract

Diethylhexyl phthalate (DEHP) is a commonly used plasticizer in the manufacture of polyvinyl chloride plastics for household and commercial use. DEHP is a ubiquitous ecocontaminant and causes developmental and reproductive problems in children and adults. After exposure, DEHP is metabolized by endogenous hydrolysis and oxidation into the primary metabolite, mono-(2-ethylhexyl) phthalate (MEHP), and the secondary metabolites, mono-(2-ethyl-5-hydroxhexyl)phthalate (5-OH-MEHP), mono-(2-ethyl-5-oxohexyl) phthalate (5-oxo-MEHP), mono-(2-ethyl-5-carboxypentyl) phthalate (5-cx-MEPP), and mono-[(2-carboxymethyl)hexyl] phthalate (2-cx-MMHP). Very few studies have been reported on the adverse effects of DEHP metabolites, and the available information indicates that the metabolites might also be equally or more active as compared to the parent compound. In the present study, induced fit docking was used for structural binding characterization of the above five DEHP metabolites with androgen receptor (AR) to predict the potential endocrine-disrupting effects of these metabolites in AR signaling. All the DEHP metabolites interacted with the ligand-binding pocket of AR forming amino-acid residue interactions, hydrogen bonding, and pi-pi interactions. The binding energy of DEHP with AR was similar to that of native ligand testosterone. The amino-acid residue interactions of DEHP metabolites had 91–100% similarity compared to that of testosterone. In addition, all the DEHP metabolites and testosterone showed a common hydrogen bonding interaction with amino-acid Arg-752 of AR. Taken together, the structural binding data in the present study suggested the potential for DEHP metabolites to disrupt AR signaling, which may lead to androgen-related reproductive dysfunction.

## 1. Introduction

Di(2-ethylhexyl) phthalate (DEHP) is a synthetic chemical prepared commercially from phthalic anhydride with an excess of 2-ethylhexanol in the presence of an acid catalyst and is the single largest plasticizer by production volume used in the world [1,2,3]. Even though the production and use of DEHP are declining due to the reported adverse effects on human health, it is forecasted to constitute more than one-third of about 10 million tons of the total plasticizer market in the world in 2024 [4,5]. The major use of DEHP is for increasing the flexibility of polyvinyl chloride plastics in which it may constitute up to 40% of the weight [2]. The main (95%) applications of DEHP are for polymer uses such as toys, footwear, shower curtains, wire and cable coating, electronic component parts, molds, wall covering, paper coating, roofing, flooring, industrial applications, medical devices, etc. In contrast, minor applications are in paints, inks, adhesives, dielectric fluids, etc. [6].

DEHP is a known endocrine-disrupting chemical (EDC) and is associated with reproductive, endocrine, and other disorders in humans such as infertility, endometriosis, thyroid hormone disruption, testicular dysgenesis syndrome, diabetes, obesity, cancer, behavioral issues, etc. [1,7,8]. DEHP has been officially prohibited for use in the manufacture of children’s toys in the United States since 2009 and has been categorized as a Group 2B human carcinogen by the United States Environmental Protection Agency. In addition, the agency has recently designated DEHP as a high-priority substance for risk evaluation based on its assessment of the unreasonable risk of injury to health and environment, including susceptible subpopulations [9]. Moreover, due to the adverse effects, DEHP is considered a priority and/or hazardous pollutant in many other countries, including Canada and the Netherlands [10]. DEHP has been designated as a dangerous chemical by the European Union and is regarded as human fertility impairing and developmental toxic (category 2) chemical [11]. The Japan Society for Occupational Health has designated DEHP as a group 1 chemical on the basis of human reproductive toxicity [12].

**Figure 1:**
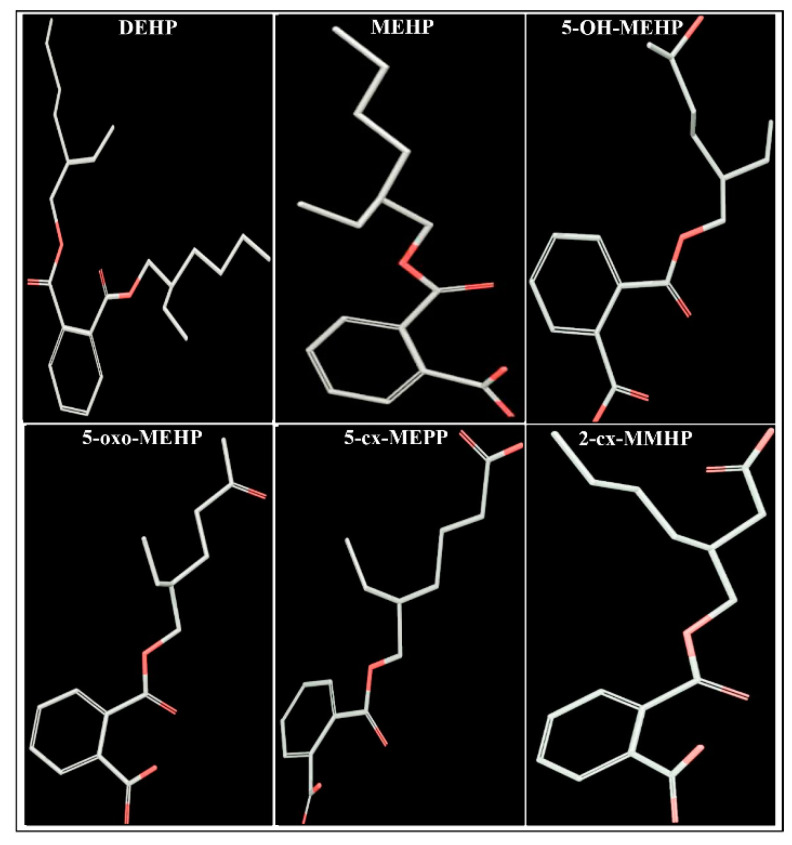
Three-dimensional representation of di-(2-ethylhexyl) phthalate (DEHP) and its five major metabolites, mono-(2-ethylhexyl)phthalate (MEHP), mono-(2-ethyl-5-hydroxyhexyl)phthalate (5-OH-MEHP), mono-(2-ethyl-5-oxohexyl)phthalate (5-oxo-MEHP), mono-(2-ethyl-5-carboxypentyl)phthalate (5-cx-MEPP), and mono-[2-(carboxymethyl)hexyl]phthalate (2-cx-MMHP).

DEHP is ubiquitously present in the environment, and major exposure from DEHP occurs through food contamination as it migrates from plastic containers and wrappings during processing and storage [2,13,14]. In one of the recent studies [15], a high majority (74%) of 72 common food products such as pork, chicken, infant foods, and other food items in the United States were contaminated with detectable levels of DEHP. Exposure also occurs through medical devices during assisted respiration, cardiopulmonary bypass, kidney dialysis, blood transfusions, incubators, etc. [16]. In addition, people working in factories that manufacture or use DEHP are subjected to significant occupational aerosol exposure. The systemic personal exposure of DEHP is measured by analysis of DEHP or its breakdown products in the urine, blood, or other body fluids [17]. After gaining access, DEHP undergoes hydrolysis in the human body to form a primary metabolite, mono-(2-ethylhexyl)phthalate (MEHP) [18]. MEHP then undergoes hydroxylation and oxidation and forms several secondary metabolites, which are excreted from the body with urine, sweat, milk, semen, and saliva [19]. Four major DEHP secondary metabolites are mono(2-ethyl-5-hydroxyhexyl)phthalate (5-OH-MEHP) and mono(2-ethyl-5-oxyhexyl)phthalate (5-oxo-MEHP), mono(2-ethyl-5-carboxypentyl)phthalate (5-cx-MEPP) and mono [2-(carboxymethyl)hexyl]phthalate (2-cx-MMHP) [18,20]. The structural illustration of the major metabolites of DEHP is given in Figure 1. After a single dose of DEHP in adult human volunteers, 5-OH-MEHP was the major metabolite in urine until 12 h and 5-cx-MEPP was the major urinary metabolite from 12–24 h, whereas, the major metabolite after 24 h was 2-cx-MMHP [20]. About 70% of the dose was excreted in urine after 24 h constituting 5-OH-MEHP (23.3%), 5-cx-MEPP (18.5%), 5-oxo-MEHP (15.0%), MEHP (5.9%), and 2-cx-MMHP (4.2%). In contrast, biomonitoring of newborns in neonatal intensive care has revealed that 5-cx-MEPP accounted for the majority (60–83%) and 5-OH-MEHP for 5–15% of the urinary DEHP metabolites [21,22].

Analyses of human body fluids such as urine [13], cord blood [23], amniotic fluid [24], follicular fluid [25], and milk and blood [26] have revealed detectable levels of DEHP metabolites. The potential adverse effects of DEHP are, in fact, thought to be more due to its metabolites; secondary metabolites are about 100 fold more embryo-toxic than MEHP [27,28]. In several other studies, reproductive and developmental problems such as preterm birth, altered reproductive hormone levels, hypospadias, cryptorchidism, anogenital distance anomalies and reduced gestational age for male babies, intellectual and motor development in children, adulthood infertility problems in men such as lower semen volume and sperm concentrations were associated with in utero exposure with DEHP and higher maternal serum levels of MEHP, 5-OH-MEHP, 5-oxo-MEHP, and 5-cx-MEPP [19,29]. In addition, higher production of reactive oxygen species, higher sperm apoptosis, and lower sperm concentration and motility in polyvinyl chloride workers was associated with higher MEHP, 5-OH-MEHP, and 5-oxo-MEHP levels in urine [19,30].

In hospitalized intensive care patients, the DEHP exposure could reach very high levels. For example, the urinary levels of 5-OH-MEHP and 5-oxo-MEHP in babies under neonatal intensive care were reported in the range of 1–5 μg/mL [21,31]. These exposures may continue for days or even weeks, thus could have severe adverse effects on the health of children and adults. The EDCs such as DEHP are thought to cause adverse effects in the body through interactions with steroid hormone receptors, hormone transport proteins, and steroid hormone enzymatic pathways [32]. Very few studies have been reported on the potential adverse effects of endogenous DEHP metabolites on human body functions. Available in vitro and structural studies on selective DEHP metabolites with androgen receptor (AR) have reported conflicting results [33,34,35,36]. Widespread environmental distribution, commercial use, and human exposure of DEHP warrant a thorough toxicological investigation of its endogenous metabolites. The current structure-based study on the interactions of DEHP metabolites with AR was done to help in understanding the potential disrupting activity of DEHP metabolites in androgen signaling. The study used molecular docking simulation to delineate the binding interactions of the five major endogenous metabolites of DEHP, i.e., MEHP, 5-OH-MEHP, 5-oxo-MEHP, 5-cx-MEPP, and 2-cx-MMHP with AR and differential comparisons with natural AR ligand testosterone for the binding pattern and the interacting residues.

## 2. Materials and Methods

The 2-dimensional structures of 6 ligands, i.e., DEHP, MEHP, 5-OH-MEHP, 5-oxo-MEHP, 5-cx-MEPP, and 2-cx-MMHP were drawn using Maestro 10.3 (Maestro, version 10.3, Schrodinger, LLC, New York, NY, USA, 2015) and this was followed by conversion of 2-dimensional structures into 3-dimensional structures (Figure 1). The structure was also searched in the PubChem compound database (https://pubchem.ncbi.nlm.nih.gov/) for identification and confirmation. The PubChem compound identities and abbreviations are presented in Table 1. For docking experiments of these ligands with AR, Schrodinger 2015 suite with Maestro 10.3 (graphical user interface) software (Schrodinger, LLC, New York, NY, USA, 2015) was used [37].

### 2.1. Protein Preparation

The database Protein Data Bank (PDB; http://www.rcsb.org/) was explored to obtain the crystal structure complex of human AR (PDB code: 2AM9) co-complexed with natural ligand testosterone (TST) at resolution of 1.64 Å. The crystal structure was prepared for docking analysis using the protein preparation wizard workflow of Schrodinger Glide (Schrodinger suite 2015-3; Schrodinger, LLC) as described earlier [37]. Briefly, the crystal structure of the AR complex was imported to the docking program Glide and prepared by removing the water molecules, adding the hydrogen atoms and charges, and building loops and missing side chains using the protein preparation wizard workflow, OPLS-2005 force field, and Prime 3.0 module software. Then, the optimization of hydrogen bonds was done, and finally, a geometry optimization was performed to a maximum root-mean-square deviation (rmsd) of 0.30 Å. The natural ligand TST in the crystal structure was selected for the generation of grid boxes and used for docking of the ligands.

### 2.2. Ligand Preparation

The 2-dimensional structures of DEHP metabolites were drawn using Maestro 10.3 (Maestro, version 10.3, Schrodinger, LLC), and this was followed by conversion of 2-dimensional structures into 3-dimensional structures (Figure 1), as described previously [37]. LigPrep module (Schrodinger 2015: LigPrep, version 3.1, Schrodinger, LLC) was used to prepare the DEHP ligands, and OPLS-2005 force field software was used for obtaining correct molecular geometries and ionization at biological pH 7.4.

### 2.3. Induced Fit Docking

Schrodinger’s Induced Fit Docking (IFD) module was utilized for experiments on docking simulations of AR with DEHP and its 5 metabolites. The prediction of accurate conformational changes in protein upon ligand binding was an issue that needed to be addressed to simulate the ligand-binding more precisely. The IDF method addressed this problem to some extent as it induced flexibility in the protein ligand-binding pocket during the docking experiment. The methodology of IFD has been described previously [37]. Briefly, DEHP metabolites were incorporated as starting geometries into the IFD software, which had the calculating capability of sampling the minor changes in the backbone structure as well as robust conformational changes in the side chains [38]. Docking of the ligand occurred into an ensemble of the binding protein conformations during the performance of a softened-potential docking in the first IFD stage. Subsequently, complex minimization for the highest-ranked pose was performed where ligand, as well as the binding sites, were free to move.

### 2.4. Binding Affinity Calculations

The ligand-binding affinity calculations of above-indicated phthalate plasticizer (DEHP) and its major metabolites for AR were performed using the Prime module of Schrodinger 2015 with molecular mechanics generalized born-surface area (MMGB-SA) function as described [37]. The MMGB-SA function was one of the best methodologies to be pursued and was a middle ground between the fast but inaccurate docking simulations and the free-energy perturbation, which gave the best results but it was very costly from a computational time perspective.

## 3. Results

**Figure 2:**
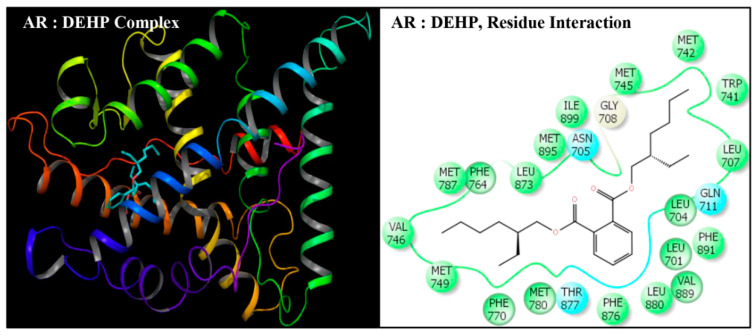
Ribbon diagram representing the docking pose of human androgen receptor (AR) with di(2-ethylhexyl) phthalate (DEHP) (left panel). The interactions of ligand (DEHP) with amino-acid residues in the binding pocket of AR (right panel).

**Figure 3:**
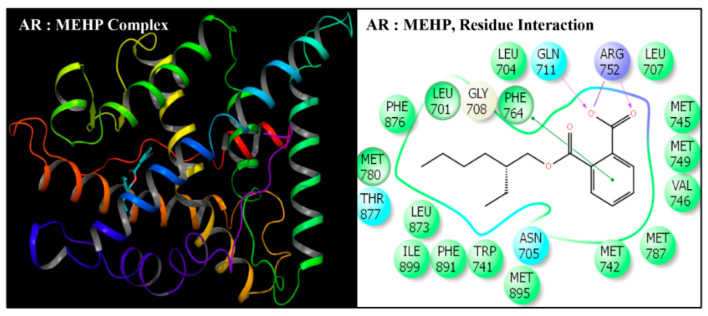
Ribbon diagram representing the docking pose of human androgen receptor (AR) with mono-(2-ethylhexyl)phthalate (MEHP) (left panel). The interactions of ligand (MEHP) with amino-acid residues in the binding pocket of AR (right panel).

**Figure 4:**
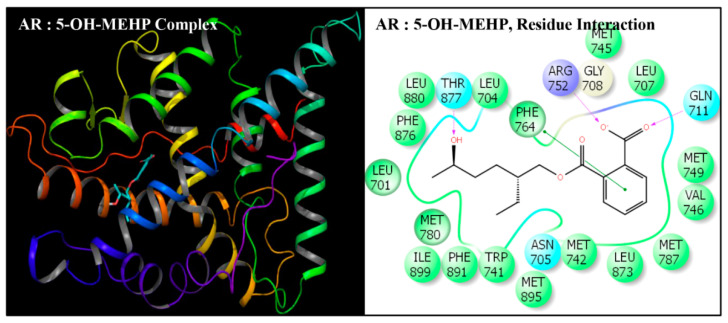
Ribbon diagram representing the docking pose of human androgen receptor (AR) with mono-(2-ethyl-5-hydroxyhexyl)phthalate (5-OH-MEHP) (left panel). The interactions of ligand (5-OH-MEHP) with amino-acid residues in the binding pocket of AR (right panel).

**Figure 5:**
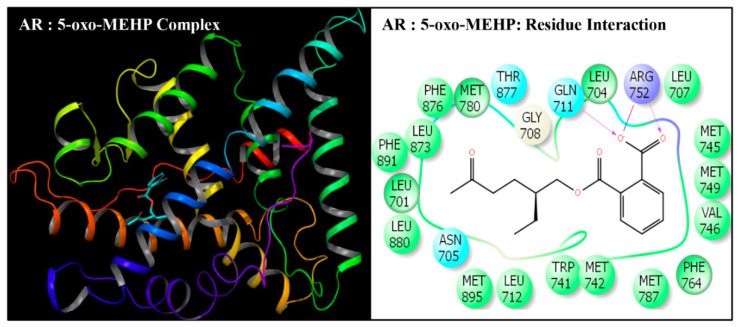
Ribbon diagram representing the docking pose of human androgen receptor (AR) with mono-(2-ethyl-5-oxohexyl)phthalate (5-oxo-MEHP) (left panel). The interactions of ligand (5-ox-MEHP) with amino-acid residues in the binding pocket of AR (right panel).

**Figure 6:**
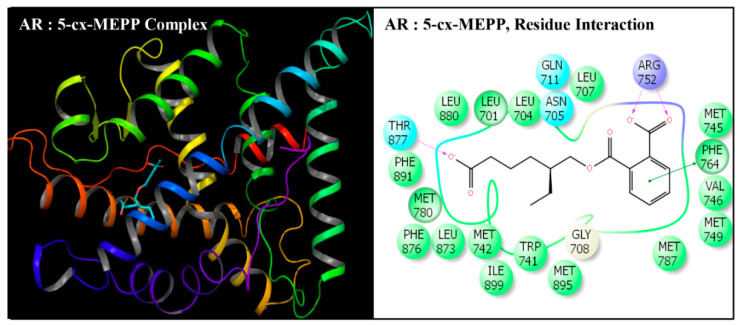
Ribbon diagram representing the docking pose of human androgen receptor (AR) with mono-(2-ethyl-5-carboxypentyl)phthalate (5-cx-MEPP) (left panel). The interactions of ligand (5-cx-MEPP) with amino-acid residues in the binding pocket of AR (right panel).

**Figure 7:**
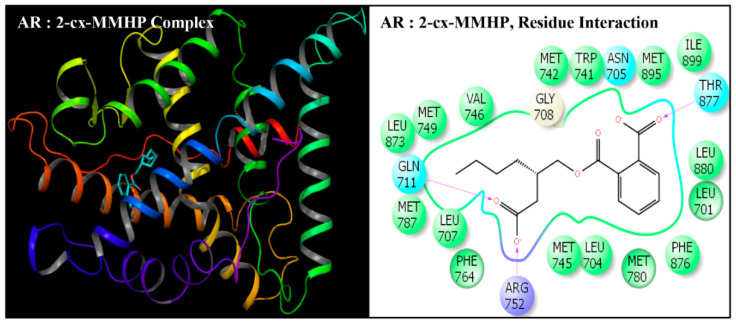
Ribbon diagram representing the docking pose of human androgen receptor (AR) with mono-[2-(carboxymethyl)hexyl]phthalate (2-cx-MMHP)(left panel). The interaction of ligand (2-cx-MMHP) with amino-acid residues in the binding pocket of AR (right panel).

DEHP and its five major endogenous metabolites, i.e., MEHP, 5-OH-MEHP, 5-oxo-MEHP, 5-cx-MEPP, and 2-cx-MMHP, were successfully subjected to docking simulation with AR. The successful accomplishment ensued into the stable placement of all the ligands in the AR ligand-binding pocket. Several interaction poses were generated for each ligand during the docking procedure in the binding domain of AR, and the most representative stable docking poses for each ligand based on the result of cumulative analysis of docking parameters, including amino acid interactions in the ligand-binding pocket, which were visualized using Maestro as graphical user interface and binding energy estimation calculated by the MMGB-SA function, which was considered for further analysis. The resulting docking poses, amino-acid interactions, and analyses are presented for each compound along with the native ligand, TST (Figure 2, Figure 3, Figure 4, Figure 5, Figure 6 and Figure 7, Table 2 and Table 3).

**Figure 8:**
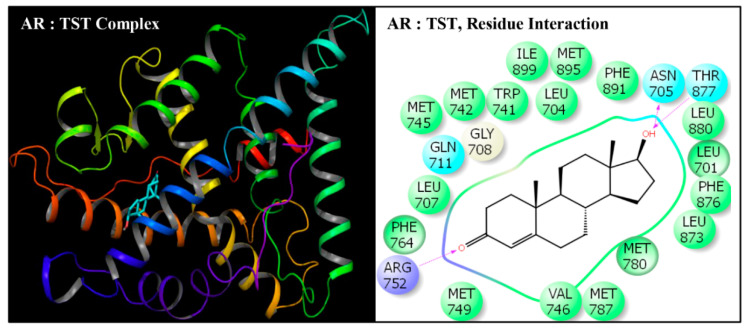
Ribbon diagram representing the docking pose of human androgen receptor (AR) with native ligand testosterone (TST) (left panel). The interactions of ligand (TST) with amino-acid residues in the binding pocket of AR (right panel).

Docking poses of six compounds (DEHP and five metabolites) in the ligand-binding domains of AR resulted in 21–23 amino-acid residue interactions (Figure 2, Figure 3, Figure 4, Figure 5, Figure 6 and Figure 7, Table 2). Further, the docking complex of native ligand, TST, displayed interactions with 22 amino-acid residues of AR (Figure 8). The list of common interacting amino-acid residues for DEHP metabolites and TST is shown (Table 3). In addition, DEHP also interacted with Phe-770 and Val-889, while 5-oxo-MEHP also interacted with Leu-712. DEHP and its five metabolites shared all the amino acid residues that interacted with native ligand TST except that four amino-acid residues, i.e., Arg-752, Leu-880, Phe-891, and Ile-899 were not shared, respectively, by DEHP, MEHP, 2-cx-MMHP, and 5-oxo-MEHP (Table 3). Overall, there was a commonality of 91–100% of residues between DEHP compounds and the native ligand (Table 2). In addition to the amino acid interactions, DEHP metabolites also formed hydrogen bonds, pi-pi bond, and salt bridge interactions with AR. The metabolites MEHP, 5-OH-MEHP, 5-oxo-MEHP, and 2-cx-MMHP each formed two hydrogen bonds with amino-acid residues Gln-711 and Arg-752. Additionally, 5-OH-MEHP and 2-cx-MMHP each formed a hydrogen bond interaction with residue Thr-877. The metabolite 5-cx-MEPP did not form a hydrogen bond with Gln-711 but formed two hydrogen bonds with Arg-752 and one hydrogen bond with Thr-877. The native ligand TST also formed three hydrogen bonds with amino-acids Asn-705, Arg-752, and Thr-877. In addition to the hydrogen bond interactions, MEHP, 5-OH-MEHP, and 5-cx-MEPP each formed a pi-pi bond with Phe-764, and MEHP and 5-oxo-MEHP each formed a salt bridge interaction with Arg-752.

The values for the Dock score, IFD score, Glide score, and binding affinity (MMGB-SA values) for all the ligands are shown (Table 2). The values for binding energy were highest for native ligand TST. The binding energy values for DEHP were close to that of TST, but the values for the five metabolites were lower (Table 2). The IFD scores were similar between five DEHP metabolites and native ligand TST. The Dock score and Glide score were highest for TST and lowest for DEHP, but progressively increased for MEHP, 5-OH-MEHP, 5-oxo-MEHP, and 5-cx-MEPP.

## 4. Discussion

The adult humans and newborns show a systemic predominance of the secondary metabolites of DEHP, which in comparison to the parent compound, remain in the system for a longer duration (20–22). In view of the toxic effects of parent compound DEHP, the present study was done to predict the potential adverse effects of the DEHP metabolites. The objective of the current research was to characterize the structure-based interactions of DEHP metabolites, i.e., MEHP, 5-OH-MEHP, 5-oxo-MEHP, 5-cx-MEPP, and 2-cx-MMHP with AR. The IFD approaches used in this study showed that DEHP and its metabolites docked deep in the ligand-binding domain of AR forming various interactions with a number of amino-acids. The molecular interactions between the ligands and the amino-acid residues in the AR ligand-binding pocket included hydrogen bonding, pi-pi, hydrophobic, salt bridge, etc. These interactions with the receptor anchored the ligands in the binding pocket. Analysis of the most stable docking pose for each ligand based on the result of cumulative analysis of docking parameters including amino acid interactions in the ligand-binding pocket of AR, and binding energy estimation revealed similar IFD score of DEHP ligands and native ligand TST together with good Glide and Dock score of the ligands indicating good strength and stability of the docking complexes. The closely similar values of binding energy between DEHP and TST also provided support for the good docking. For all the DEHP metabolites, the amino-acids interacting with AR were 95–100% common when compared with native ligand TST, which again indicated similarity in their structural and spatial conformation and, hence, manifested in docking. Further, a common hydrogen bonding interaction of DEHP metabolites and native ligand TST with amino-acid residue Arg-752 of AR also indicated a common binding mechanism of the DEHP metabolites and the native ligand. Additional hydrogen bonding interactions with amino-acid residues Gln-711 and Thr-877, pi-pi interactions with Phe-764, and salt bridge interaction with amino-acid residue Arg-752 of AR also provided support for the strength and stability of the binding of DEHP metabolites with AR. Hence, the similarity in docking and amino acid interactions between DEHP and its metabolites and native ligand TST with AR indicated the potential of DEHP metabolites for interference in the TST binding. A potential interference in binding may result in AR signaling disruption and abnormal androgen-related reproductive function.

The toxicological evaluation has been exclusively done for the parent compound DEHP, and very few studies have been conducted on DEHP metabolites, especially the secondary metabolites. Further, in vitro and in silico studies have been largely equivocal about the antagonistic activity of DEHP metabolites against AR [39,40,41]. In a recent study [34], using luciferase assays for analyzing the androgenic or antiandrogenic activity of DEHP and its important metabolites, it was shown that in contrast to the antiandrogenic activity of DEHP, the primary and secondary metabolites did not show any activity with AR. Whereas, in another recent study [35], using similar luciferase assays, neither DEHP nor its major primary and secondary metabolites showed any antagonistic activity against AR. This is in contrast to the structural binding results in the present study in which DEHP and its metabolites interacted with AR and were predicted to exert antagonistic effects. However, our results are supported by a study using yeast androgen bioassay (XenoScreen YES/YAS assay) in which metabolite MEHP was shown to exhibit anti-androgenic activity [36]. These inconsistent results were also observed in in silico docking studies. In one study [33], DEHP and its primary metabolite MEHP were reported to efficiently bind to human AR supporting our docking results in the present study. In addition, Leu-704, Asn-705, Gln-711, Arg-752, Phe-764, and Thr-877 were found as the crucial amino-acid residues in the ligand-binding pocket of AR in both the reported study and our study. In contrast, in another docking study, DEHP and its primary and secondary metabolites (MEHP, 5-OH-MEHP, 5-oxo-MEHP, 5-cx-MEPP, and 2-cx-MMHP) were reported to not interact with AR, and the study suggested a very low possibility of their antiandrogenic activity [35]. The differences in the results are not known. The direct in vivo toxicological experiments in laboratory animals on 5-OH-MEHP, 5-oxo-MEHP, 5-cx-MEPP, and 2-cx-MMHP are not available. In view of the previous equivocal results and lack of direct in vivo studies on the metabolites, further in vitro, in vivo, and structural studies on adverse effects of DEHP metabolites on androgen signaling are recommended to explore their endocrine-disrupting mechanisms. Special consideration must be given to DEHP metabolites in view of the ubiquitous nature of DEHP as even a weak significant association with adverse outcomes may be profoundly relevant for exposed populations. Computational models have very important relevance in the fast predictive assessment of the toxicity of environmental chemicals to human systems. It might be important to consider a combination of computational modelings such as pharmacophore screening, molecular docking, and MD simulations together with experimental assays for a holistic approach for more reliable prediction of AR disrupting risk of environmental compounds such as DEHP and its metabolites.

Epidemiological studies have associated DEHP as a cause for testicular dysgenesis syndrome manifested by infertility, genital developmental problems such as hypospadias, cryptorchidism, etc., and testicular cancer in exposed men [42,43]. Unborn and newborn babies being at maximum risk of adverse effects due to their immature metabolism and excretory systems. In this regard, some of the adverse effects of prenatal exposure are preterm birth, anogenital distance problems, low birth weight, low rate of weight gain, and effects on the femur, birth, head, biparietal, and abdominal dimensions in male and female babies [44]. DEHP exposure during gestation increased the risk of DNA methylation problems interfering in gene expression and adverse health conditions during childhood [45]. In addition, prenatal exposure was associated with aggressive behavior, defiance, and poor attention in children [46]. Precocious development such as early menarche of girls between 6–14 years of age and delayed menarche such as poor hair and testis development in boys have been associated with higher urinary MEHP levels [47]. In other studies, semen and urine levels of MEHP, 5-OH-MEHP, 5-oxo-MEHP, and DEHP metabolites excreted as percentage of MEHP predisposed to a low volume of semen, abnormal morphology, low motility, and lower testosterone levels in infertile men [48]. DEHP metabolites were also associated with high sperm DNA stainability indicating sperm immaturity [19]. In Japan, gestational circulatory MEHP levels were associated with lower concentrations of testosterone, progesterone, estradiol, cortisol, cortisone, insulin-like factor 3 and inhibin-B, and a lower ratio of glucocorticoid/adrenal androgen but higher concentrations of dehyroepiandrostenedione (DHEA) and a higher ratio of DHEA/androstenedione in cord blood of male newborns [49,50]. A recent study reported that in vitro exposure of human spermatozoa to DEHP and MEHP caused increased calcium in sperms and increased tyrosine phosphorylation. However, the significance of these effects is yet unknown [51].

In experimental studies on rats, pre and postnatal DEHP exposure have been associated with intrauterine mortality, impaired testicular development, teratogenicity, reduced anogenital distance, nipple retention, Leydig cell hyperplasia, hormone (especially testosterone) imbalance, and other reproductive system developmental problems even with relatively lower doses [52,53]. Particularly in male newborn rats, prenatal DEHP exposure causes congenital disabilities such as typical male dysgenesis or phthalate syndrome showing testis tubule atrophy, anogenital, epididymal, testicular, vas deferens, and external genitalia anomalies [54,55]. In adult male rats, DEHP is gonadotoxic and leads to testis atrophy, reduced litter size, lower sperm concentration, lower sperm motility, high sperm abnormalities, lower sperm fertilizing ability, zinc depletion, lower DNA replication, increased apoptosis, increase oxidative stress [56].

## 5. Conclusions

The current study prioritized the prediction of potential AR disrupting effects of DEHP metabolites by in silico structural interactions of the major endogenous hydroxylated and oxidative metabolites (MEHP, 5-OH-MEHP, 5-oxo-MEHP, 5-cx-MEPP, and 2-cx-MMHP) of DEHP with AR. The structural interaction data showed that DEHP and the five major metabolites have relatively strong binding affinities for AR. The binding energy values of DEHP were similar to that of native ligand TST. A very high (95–100%) similarity was found in interacting AR amino-acid residues among DEHP metabolites and TST. In addition, hydrogen bonding between all the DEHP metabolites and amino-acid Arg-752 of AR was also common with native ligand TST. Taking into consideration the reported adverse effects of DEHP and similarity in docking and amino acid interactions among DEHP metabolites and the native ligand TST with AR, the data suggest a potential disrupting effect of DEHP metabolites on AR signaling, which may lead to abnormal androgen-related reproductive function. From public health perspectives, this study underscores the need for reduced use of plastic materials containing DEHP in industrial and commercial applications and spreading public awareness of their health hazards, especially in vulnerable pregnant women and newborn children. Further studies, especially computational modeling and in vitro and in vivo experiments, are recommended for predicting and assessing the AR-disrupting risk of environmental compounds such as DEHP and its metabolites.

## Figures and Tables

**Table 1:** Ligand name, common abbreviations, PubChem IDs and Chemical Abstract Service Registration Number (CAS No.) of di(2-ethylhexyl) phthalate and its five major endogenous metabolites.

Serial No.	Name	Abbreviation	PubChem ID	CAS No
1	Di(2-ethylhexyl)phthalate	DEHP	8343	117-81-7
2	Mono-(2-ethylhexyl)phthalate	MEHP	20393	4376-20-9
3	Mono-(2-ethyl-5-hydroxyhexyl)phthalate	5-OH-MEHP	170295	40321-99-1
4	Mono-(2-ethyl-5-oxohexyl)phthalate	5-oxo-MEHP	119096	40321-98-0
5	Mono-(2-ethyl-5-carboxypentyl)phthalate	5-cx-MEPP	148386	40809-41-4
6	Mono-[2-(carboxymethyl)hexyl]phthalate	2-cx-MMHP	187353	82975-93-7

**Table 2:** The number of interacting residues, number and percentage of residues common with native ligand testosterone (TST), Induced Fit Docking (IFD) Score, Dock score, Glide score, and binding affinity values (MMGB-SA values) of di(2-ethylhexyl)phthalate (DEHP), mono-(2-ethylhexyl)phthalate (MEHP), mono-(2-ethyl-5-hydroxyhexyl)phthalate (5-OH-MEHP), mono-(2-ethyl-5-oxohexyl)phthalate (5-oxo-MEHP), mono-(2-ethyl-5-carboxypentyl)phthalate (5-cx-MEPP), and mono-[2-(carboxymethyl)hexyl]phthalate (2-cx-MMHP) and native ligand, TST, after IDF with human androgen receptor (AR).

Serial No.	Ligand	Number of Interacting AR Residues	Number of Interacting Residues Common with TST (%)	IFD Score	Docking Score (kcal/mol)	Glide Score (kcal/mol)	MMGB-SA (kcal/mol)
1	DEHP	23	21 (95%)	−573.86	−9.17	−9.17	−136.12
2	MEHP	21	21 (95%)	−573.30	−9.42	−9.42	−109.19
3	5-OH-MEHP	22	22(100%)	−573.33	−9.65	−9.65	−107.50
4	5-oxo-MEHP	22	20 (91%)	−574.45	−9.73	−9.73	−105.80
5	5-cx-MEPP	21	22 (100%)	−575.94	−11.69	−11.69	−92.03
6	2-cx-MMHP	21	21 (95%)	−574.48	−10.41	−10.41	−90.60
7	TST	22	22 (100%)	−577.85	−12.84	−12.84	−152.11

**Table 3:** Interacting amino-acid residues in the binding pocket of human androgen receptor that were common among natural ligand, testosterone, and di(2-ethylhexyl) phthalate (DEHP) and its five major metabolites, mono-(2-ethylhexyl)phthalate (MEHP), mono-(2-ethyl-5-hydroxyhexyl)phthalate (5-OH-MEHP), mono-(2-ethyl-5-oxohexyl)phthalate (5-oxo-MEHP), mono-(2-ethyl-5-carboxypentyl)phthalate (5-cx-MEPP), and mono-[2-(carboxymethyl)hexyl]phthalate (2-cx-MMHP).

Serial No.	Interacting Residue	Serial No.	Interacting Residue
1	Leu-701	12	Arg-752 *
2	Leu-704	13	Phe-764
3	Asn-705	14	Met-780
4	Leu-707	15	Met-787
5	Gly-708	16	Leu-873
6	Gln-711	17	Phe-876
7	Trp-741	18	Thr-877
8	Met-742	19	Leu-880 ^$^
9	Met-745	20	Phe-891 ^&^
10	Val-746	21	Met-895
11	Met-749	22	Ile-899 ^#^

Amino-acid residues indicated by superscripts were not shared DEHP (*), MEHP (^$^), 2-cx-MMHP (^&^) and 5-oxo-MEHP (^#^).
